# Nutritional status and disease severity in children acutely presenting to a primary health clinic in rural Gambia

**DOI:** 10.1186/s12889-019-6959-y

**Published:** 2019-05-30

**Authors:** Henry Mark, Jasper V. Been, Bakary Sonko, Abdoulie Faal, Mohammed Ngum, Jahid Hasan, Andrew M. Prentice, Stefan A. Unger

**Affiliations:** 1 MRC Unit The Gambia, Atlantic Boulevard, Fajara, P.O. Box 273, Banjul, The Gambia; 2 Division of Neonatology, Department of Paediatrics, and Department of Obstetrics and Gynaecology, Erasmus MC – Sophia Children’s Hospital, University Medical Centre Rotterdam, Rotterdam, The Netherlands; 3 Department of Public Health, Erasmus MC, University Medical Centre Rotterdam, Rotterdam, The Netherlands; 4 Centre for Medical Informatics, Usher Institute of Population Health Sciences and Informatics, The University of Edinburgh, Edinburgh, UK; 5 MRC International Nutrition Group, London School of Hygiene & Tropical Medicine, Keppel Street, London, WC1E 7HT UK; 6 Department of Child Life and Health, University of Edinburgh, 20 Sylvan Place, Edinburgh, EH9 1UW UK

**Keywords:** Electronic medical records, Nutrition status, Infectious disease, Primary health care, Sub-Saharan Africa

## Abstract

**Background:**

Accurate and timely data on the health of a population are key for evidence-based decision making at both the policy and programmatic level. In many low-income settings, such data are unavailable or outdated. Using an electronic medical records system, we determined the association between nutritional status and severe illness and mortality among young children presenting to a rural primary health care facility in the Gambia.

**Methods:**

Clinical data collected over five years (2010–2014) on children aged under 60 months making acute visits to a primary health care clinic in the rural Gambian district of Kiang West were retrospectively extracted from the medical records system. Generalised estimating equation models were used to investigate associations between nutritional status and illness severity, accounting for repeat visits, gender, age and access to transport to the clinic. The Population Attributable Fraction (PAF) was used to determine the proportion of severe illness likely attributable to different grades of malnutrition.

**Results:**

3839/5021 (77%) children under 60 months of age living in Kiang West presented acutely to the clinic at least once, yielding 21,278 visits (47% girls, median age 20.2 months (Interquartile Range (IQR) 23.92 months)) and 26,001 diagnoses, 86% being infectious diseases. Severe illness was seen in 4.5% of visits (961/21,278). Wasting was associated with an increased risk of severe illness in a dose-dependent manner, (‘WHZ < -1’ adjusted Odds Ratio (aOR) 1.68, 95% CI:1.43–1.98, *p* < 0.001, ‘WHZ <-2 and ≥-3’ aOR 2.78, 95% CI:2.31–3.36, p < 0.001 and ‘WHZ < -3’ aOR 7.82, 95% CI:6.40–9.55, p < 0.001) the PAF for wasting (WHZ < -2) was 0.21 (95% CI: 0.18–0.24). Stunting, even in the most severe form (HAZ < -3), was not significantly associated with severe illness (aOR 1.19 95% CI:0.94–1.51) but was associated with a significantly increased risk of death (aOR 6.04 95% CI:1.94–18.78).

**Conclusion:**

In this population-based cohort of young children in rural Gambia, wasting was associated with disease severity in a dose-dependent manner. Further research is needed into strategies to identify and reach these children with effective interventions to improve their nutritional status.

**Electronic supplementary material:**

The online version of this article (10.1186/s12889-019-6959-y) contains supplementary material, which is available to authorized users.

## Background

Quality health information is a prerequisite for improving clinical practice, informing public health approaches and guiding health policy. Health information systems draw data from a range of sources including: epidemiological studies, birth and death registers and health facility records. John Fry’s work on common diseases provides a historical example of how primary care data can provide a window into the health of a population [1]. Migration from paper to electronic health records has further facilitated the utilisation of health facility data [2], which can be used for auditing, quality improvement and epidemiological purposes. In the absence of well-designed longitudinal surveys–which are generally required to quantify the burden of infectious disease morbidity [3]–health facility data can provide useful insights into population morbidity profiles. Given the expense and level of technical capacity to manage such systems, few low-income countries have implemented one [4–6].

There are limited examples in the published literature where routinely collected primary care data from sub-Saharan African countries has been used for epidemiological purposes (see additional file 1, section 1 for literature search criteria). A well-defined cohort study in South Africa used primary care data to describe the disease profiles of 1357 children under 5 years presenting to primary care clinics between 2006 and 2007 [7]. Data from 4026 prescriptions given on the ‘Phelophepa’ primary care train in South Africa were also used to determine prescribing characteristics [8]. However, we are not aware of any analysis using primary care data to describe the morbidity profiles of a population in a West African setting.

This is the first study utilising clinical data collected via an electronic medical records system for all children under 60 months presenting to a primary health care facility in the Kiang West district, The Gambia [9]. We sought to provide an insight into illness severity and mortality of clinic presentations in relation to nutritional status of children under 60 months making acute presentations to the clinic.

## Methods

### Study site and database

This study utilises data on children under 60 months of age presenting to the Keneba primary health care clinic in The Gambia, collected over a five-year period (1st January 2010 - 31st December 2014). The clinic serves the population of Kiang West, a rural Gambian district with 36 villages and a population of ~ 14,000. Annual in- and out-migration for Kiang West stands at 90 and 89 per 1000 population respectively [9]. Free health care is provided to all Kiang West residents by the Medical Research Council (MRC) Unit, The Gambia. Data were collected via the demographic surveillance system (DSS) and the Keneba Electronic Medical Records System (KEMReS) capturing detailed information on all clinic presentations; a platform previously described by Hennig and Colleagues [9]. Clinic visits were classified into: Non-acute (child welfare visits as part of childhood surveillance and follow-up visits scheduled by clinic staff;), and acute (self-referred and emergency visits as assessed by nursing staff). Unless otherwise stated, analyses refer to acute clinic visits only throughout the text.

On presentation, vital signs and anthropometric measurements were taken by trained clinic personnel (see Hennig et al. [9] for details of all data captured). Length (age < 12 m) or height (age ≥ 12 m) was measured using a Harpenden Infantometer length board (Holtain Ltd) or stadiometer (Leicester Height Measure; model MKII) respectively. Weight was measured using digital scales (Seca, model 336; sensitivity 0.01 kg for infants or Tanita HD-305 scale; sensitivity 0.1 kg, for older children). The attending doctor recorded presenting complaints and physical findings. Disease classifications followed the International Classification of Diseases (ICD)-10 listings, prescribed medications were linked to each patient episode.

### Data extraction, cleaning and variables

The cumulative under 60-month-old population in Kiang West between 2010 and 2014 was derived from the Kiang West DSS, dates of death were also extracted. Clinic data were extracted from the KEMReS database (see Additional file 1, section 2 for full details of data extraction).

Clinical severity was retrospectively assessed using five criteria: 1) history and examination using the severe illness criteria set by the integrated management of childhood illness [10] 2) physician’s interpretation if a child looked acutely ill, 3) the need for parenteral treatment, 4) the need for admission or referral, and 5) a paediatric advanced warning score (PAWS) [11] greater than 2. Each criteria fulfilled added one point to a child’s score, with an overall score of 2 or more indicating severe illness (for full details and rational see Additional file 1, section 3 and Rees et al. [11]).

The WHO 2006 child growth standards were used to calculate Height-for-Age (HAZ), Weight-for-Height (WHZ) and Weight-for-Age (WAZ) Z-scores [12]. Data were excluded if they fell outside of a biologically plausible range, taken to be greater than +/− 5 Z-scores. Variables were created for wasting (WHZ) and stunting (HAZ), categorised as ‘at risk’ (Z-score < − 1 and ≥ − 2) ‘moderate’ (Z-score < − 2 and ≥ − 3) and ‘severe’ (Z-score < − 3) [13]. ‘Wasting’ or ‘Stunting’ refers to the sum of both ‘moderate’ and ‘severe’ (i.e. Z-score < − 2). Based on previous estimates on the average duration of untreated severe acute malnutrition [14], we restricted our analysis on the association between wasting and mortality to children for whom WHZ data were available within the 101 days preceding death, including acute and non-acute presentations. Access to transport was defined based on village of residence within Kiang West, with those living in the villages of Keneba, Manduar or Kantong Kunda having access to free weekly transport to the health facility.

### Statistical analysis

Only data from acute clinic visits were included for presenting complaints, diagnoses and illness severity. Welch’s unequal variances t-test was used to determine if nutritional status differed between those classified as severely ill and those not. All acute presentations were included in the analysis, regardless if they were repeat visits by the same individual.

Generalised estimating equation models (to account for the multiple visits by the same individuals) with a log link, binomial distribution and utilising the unique child identification as a cluster variable (STATA IC 14) were used to examine the association between nutritional status (categorical variable), and severe illness (dichotomous variable) and death (dichotomous variable) adjusting for: age (continuous variable), gender (dichotomous variable) and access to transport to the clinic (dichotomous variable). The proportions of severe illness attributable to different grades of malnutrition were calculated as the Population Attributable Fraction (PAF) [15].

### Ethics approval

The Joint Gambian Government/MRC Gambia Ethics Committee granted approval for the collection and use of anonymised demographic surveillance data in Kiang West in 1981. This approval was extended to the use of routine clinical data collected at the MRC Keneba clinic from 2009 and covered the period of data collection (2009–2014) used in this study.

## Results

Between 1st January 2010 and 31st December 2014, the cumulative under 60 months old population of Kiang West was 5021. Of these, 3839 (77%) made at least one acute visit to the Keneba clinic, with a total of 21,278 visits (median of 5 visits per individual over the reporting period (IQR = 7). In addition, 7452 non-acute visits (follow-up and welfare clinic appointments) were made by 1495 children, of those only 39 children made non-acute visits to the clinic. The median age at presentation for acute clinic visits was 20.2 m (IQR = 25.9) with 47% of visits made by girls (Table 1).

**Table 1:** Demographics of children under 60 months presenting to the Keneba clinic and of all children under 60 months to die in Kiang West over the reporting period (January 2010 to December 2014)

	All (*n* = 21,278)	Non-severe (*n* = 20,317)	Severe (*n* = 961)	All deaths (*n* = 160)^a^	Deaths: no prior clinic visit (*n* = 110)^a^	Deaths: prior clinic visit (*n* = 50)	*p*-value (death no visit vs death with visit)	N = 21,278 (%)^b^
Median age at visit/death, months (IQR)	20.23 (23.92)	20.47 (26.12)	15.67 (18.30)	0.10 (1.03)	0.13 (4.11)	12.72 (21.70)	< 0.001	21,278 (100%)
Presentations / deaths by neonates (%)	323 (1.5%)	283 (1.4%)	40 (4.2%)	78 (48.1%)	69 (62.7%)	7 (14.0%)	< 0.001	21,278 (100%)
Presentations / deaths by infants (%)	6606 (31.0%)	6243 (30.7%)	362 (37.7%)	121 (74.7%)	95 (86.4%)	24 (48.0%)	< 0.001	21,278 (100%)
Presentations / deaths by girls (%)	10,089 (47.4%)	9666 (47.6%)	423 (44.0%)	75 (46.9%)	51 (46.2%)	24 (48.0%)	0.85	21,278 (100%)
Median mothers age at visit / death, years (IQR)	30.9 (11.1)	30.8 (11.1)	31.5 (11.1)	–	–	28.2 (10.9)	–	20,099 (94.5%)
Median birth order at visit / death (IQR)	4 (4)	4 (4)	4 (5)	–	–	4 (4)	–	20,099 (94.5%)
Median distance (kilometre) to clinic (IQR)	5.3 (11.6)	5.3 (11.6)	10.5 (19.0)	16.8 (13.5)	21.0 (12.4)	13.2 (8.8)	0.02	21,278 (100%)
Access to free transport to clinic (%)	11,692 (55.0%)	11,292 (44.4%)	400 (41.6%)	21 (13.1%)	15 (13.6%)	6 (11.5%)	0.78	21,278 (100%)
Mother education level								17,282 (81.2%)
Data unavailable	3966 (18.8)	3848 (19.0)	148 (15.4)	–	–	13 (26.0)	–	
No formal education	13,728 (64.5)	13,061 (63.4)	667 (69.5)	–	–	27 (54.0)	–	
Primary	2208 (10.4)	2101 (10.3)	107 (11.1)	–	–	0 (0.0)	–	
Lower secondary	171 (0.8)	166 (0.8)	5 (0.5)	–	–	3 (6.0)	–	
Upper secondary	990 (4.6)	962 (4.7)	28 (2.9)	–	–	0 (0.0)	–	
College and university	185 (0.9)	179 (0.9)	6 (0.6)	–	–		–	

^a^ Data on mothers age, mother’s education level and child’s birth order were unavailable for children who did not attend the clinic prior to death

^b^ Total number of data points available for each variable (% of visits for which data are available)

A total of 160 deaths in children under 60 m were recorded in Kiang West over the period (median age at death was 1.3 m, IQR = 12.3) representing an under 60 m mortality rate of 51 deaths per 1000 live births. Of these, 31% (50/160) presented to the clinic at any point prior to death, with a median time of 10 days (IQR = 151) between final presentation to the clinic and death. Table 1 shows the characteristics of both those who did and did not attend the clinic prior to death. Children were less likely to present to the clinic at any point prior to death if death occurred in the neonatal period (OR = 0.10, 95% CI = 0.04–0.24 *p* < 0.001) or infancy (OR = 0.15, 95% CI = 0.07–0.32 p < 0.001). The distance to the clinic for those who did not present prior to death (median = 21 km (IQR = 12) was significantly greater than for those who did present (median = 13 km (IQR = 9, *p* = 0.02), irrespective of age at death. (Table 1).

The median number of presenting complaints per visit was two (IQR = 2) with 1242/21,278 visits having no complaint recorded. The most common complaints were fever and cough, noted in 73% (15,478/21,278) and 52% (10,960/21,278) of visits respectively. Of the 26,001 diagnoses (median of 1 diagnosis per visit (IQR = 1), 22,456 (86%) were for infectious diseases. Antibiotics were prescribed in 67% (14,327/21,278) of all visits, (for full details of presenting complaints, diagnoses and prescriptions see Additional file 1, section 4), including to 31% of children with common cold as their sole diagnosis.

In 4.5% of visits (961/21,278) patients were classified as being severely ill. Disease categories differed significantly between those with and without severe illness (Table 2). Pneumonia was the most common diagnosis in patients with severe illness (287/961, 30%) (see additional file 1, section 4). The risk of severe disease declined with age (Table 3). Of the 50 children to present to the clinic prior to death 72% (18/25) were severely ill at their last clinic visit.

**Table 2:** Classification of 24,671 diagnoses made during 21,278 acute clinic visits and 1330 diagnoses made during 961 severe illness presentations, and types of infectious diseases diagnoses in severe and non-severe presentations

	Non-severe	Severe	χ^2^ *P-value*
Diagnosis Categories for 26,001 diagnoses made during acute visits			
Well	407/24,671 (1.7%)	0/1330 (0%)	< 0.001
Infection	21,428/24,671 (86.8)	1028/1330 (77.2%)	
Injury	372/24,671 (1.5%)	14/1330 (1.1%)	
Nutritional	663/24,671 (2.7%)	188/1330 (14.2%)	
Other	1801/24,671 (7.3%)	100/1330 (7.5%)	
Type of infectious disease for 22,456 infectious disease diagnoses			
Respiratory	10,212/21,428 (47.7%)	427/1028 (41.6%)	< 0.001
Skin	4314/21,428 (20.1%)	59/1028 (5.7%)	
Diarrhoea	4214/21,428 (19.7%)	219/1028 (21.3%)	
Malaria	86/21,428 (0.4%)	37/1028 (3.6%)	
Other	2602/21,428 (12.1%)	286/1028 (27.8%)

**Table 3:** Nutritional status of children during acute visits to the clinic according to illness severity

	All acute visits (N = 21,278)	Non-severe (N = 20,317)	Severe illness (N = 961)	*P* value (severe vs non-severe)	Last visit before death (N = 50)
Mean WHZ (SD)	−0.70 (1.20)	−0.66 (1.17)	−1.48 (1.47)	< 0.001^a^	−1.45 (1.65)^c^
All wasting	2405/20,478 (11.7%)	2114/19,554 (10.8%)	291/924 (31.5%)	< 0.001^b^	11/30 (36.7%)^c^
Moderate wasting	1923/20,478 (9.4%)	1761//19,554 (9.0%)	162/924 (17.5%)	< 0.001 ^b^	4/30 (13.3%)^c^
Severe wasting	482/20,478 (2.4%)	353/19,554 (1.8%)	129/924 (14.0%)	< 0.001 ^b^	7/30 (23.3%)^c^
Mean HAZ (SD)	−1.21 (1.30)	−1.21 (1.28)	−1.34 (1.59)	0.001^a^	−2.17 (1.27)
All stunting	4389/20,545 (21.4%)	4143/19,614 (21.1%)	246/931 (26.4%)	< 0.001 ^b^	27/50 (54.0%)
Moderate stunting	3345/20,545 (16.3%)	3179/19,614 (16.2%)	166/931 (17.9%)	0.17 ^b^	13/50 (26.0%)
Severe stunting	1043/20,545 (5.1%)	964/19,614 (4.9%)	79/931 (8.5%)	< 0.001 ^b^	14/50 (28.0%)
Mean WAZ (SD)	−1.21 (1.08)	−1.19 (1.06)	−1.73 (1.26)	< 0.001^a^	−2.49 (1.55)
All underweight	4519/20605 (21.9%)	4164/19,686 (21.2%)	355/919 (38.6%)	< 0.001 ^b^	28/48 (60.9%)
Moderate underweight	3529/20605 (17.1%)	3321/19,696 (16.9%)	208/919 (22.6%)	< 0.001 ^b^	10/48 (21.7%)
Severe underweight	990/20605 (4.8%)	843/19,686 (4.3%)	147/919 (16.0%)	< 0.001 ^b^	18/48 (39.1%)

^a^ P-value for Welch’s unequal variances t-test

^b^ χ^2^ P-value

^c^ *N* = 30 for WHZ and wasting

Data on wasting (WHZ) were available for 20,478/21,278 visits (96%) (outside plausible range *n* = 51, missing *n* = 749) and on stunting (HAZ) for 20,545/21,278 visits (97%) (outside plausible range *n* = 122, missing *n* = 611) (Table 3, for demographics of children with and without missing anthropometric data see Additional file 1, section 5). Children over 12 m old had significantly higher prevalence of mild, moderate and severe stunting than infants, whereas the prevalence of wasting was similar between the age groups (additional file 1, section 6). The coexistence of stunting in children classified as wasted was 33% (788/2405) and the presence of wasting in children classified as stunted was 18% (788/4389). Nutrition-related diagnoses accounted for 14% (188/1330) of all severe illness diagnoses, compared with 3% (663/24,670) of non-severe illness (Table 2): OR 5.97 95% CI:5.02–7.09, *p* < 0.001. Both mean HAZ and WHZ where significantly lower in children with severe illness (Table 3). Of the 50 children who presented to the clinic prior to death 33 (66%) presented in the 101 days preceding death, with WHZ data available for 30 of these individuals (outside plausible range *n* = 1, missing *n* = 2). The mean WHZ of these 30 children at their last clinic visit before death (mean = − 1.45, SD = 1.65) was significantly lower than that of children during other acute clinic visits (mean = − 0.70, SD = 1.15; W = 3.68, *p* = 0.04).

Wasting was significantly associated with a higher risk of severe illness in a dose-dependent manner (‘risk of wasting’ aOR 1.68, 95% CI:1.43–1.98, p < 0.001, ‘moderate wasting’ aOR 2.78, 95% CI:2.31–3.36, p < 0.001 and ‘severe wasting’ aOR 7.82, 95% CI:6.40–9.55, p < 0.001). The association between different grades of wasting was observed for all specific infectious diseases, with the exception of severe bronchiolitis (Table 4). The fraction of severe illness attributable to severe wasting, calculated as PAF, was 0.11 (95% CI:0.09–0.13) and for wasting overall 0.21 (95% CI:0.18–0.24). Including those at risk of wasting increased the PAF to 0.35 (95% CI:0.29–0.40). Stunting on the other hand, even in its severe form was not significantly associated with illness severity. Both severe stunting (aOR 6.04, 95% CI: 1.94–18.78, P = < 0.001) and severe wasting (aOR 9.33, 95% CI:3.39–25.70, *P* < 0.001) at the last clinic presentation were however associated with a significantly increased risk of subsequent death (Table 4).

**Table 4:** Determinants of severe illness, overall and by specific infectious diseases, during 21,278 clinic visits by children under 5 years to the MRC Keneba clinic, and the association between these determinants and mortality in 50 children

	All severe illness N = 961			Severe intestinal infections *N* = 183			Severe LRTI *N* = 348			Severe pneumonia *N* = 287			Severe bronchiolitis *N* = 66			Death N = 50		
	OR (95% CI)	aOR (95% CI)	P	RR (95% CI)	aOR (95% CI)	P	RR (95% CI)	aOR (95% CI)	P	RR (95% CI)	aOR (95% CI)	P	RR (95% CI)	aOR (95% CI)	P	RR (95% CI)	aOR (95% CI)	P
Female	0.87 (0.75–1.00)	0.96 (0.83–1.11)	0.58	0.61 (0.44–0.83)	0.76 (0.55–1.06)	0.10	1.03 (0.81–1.31)	1.05 (0.82–1.35)	0.69	1.03 (0.80–1.32)	0.84 (0.62–1.14)	0.26	0.87 (0.52–1.46)	0.88 (0.51–1.49)	0.63	1.02 (0.59–1.79)	1.81 (0.86–3.78)	0.12
Age (months)^a^	0.98 (0.98–0.99)	0.98 (0.98–0.99)	< 0.001	0.96 (0.95–0.97)	0.96 (0.94–0.97)	< 0.001	0.97 (0.96–0.98)	0.97 (0.96–0.98)	< 0.001	0.98 (0.97–0.99)	0.98 (0.97–0.99)	< 0.001	0.91 (0.88–0.94)	0.92 (0.89–0.94)	0.00	0.95 (0.92–0.97	0.93 (0.87–0.99)	0.03
Risk stunting HAZ - ≤ 1 & > −2)	0.91 (0.78–1.07)	0.93 (0.79–1.08)	0.34	0.80 (0.56–1.15)	0.83 (0.57–1.20)	0.31	0.79 (0.61–1.03)	0.85 (0.66–1.10)	0.22	0.89 (0.67–1.18)	0.81 (0.57–1.16)	0.26	0.48 (0.27–0.58)	0.68 (0.38–1.24)	0.21	1.81 (0.79–4.14)	1.82 (0.71–4.67)	0.21
Moderate stunting (HAZ ≤ -2 and > − 3)	1.11 (0.92–1.34)	1.01 (0.83–1.22)	0.95	1.04 (0.68–1.59)	0.86 (0.56–1.33)	0.50	0.84 (.61–1.16)	0.85 (0.61–1.19)	0.35	1.02 (0.72–1.43)	0.93 (0.62–1.41)	0.74	0.33 (0.12–0.86)	0.54 (0.21–1.40)	0.20	3.73 (1.60–8.68)	1.89 (0.56–6.38)	0.31
Severe stunting (HAZ ≤ -3)	1.62 (1,26–2.08)	1.19 (0.94–1.51)	0.15	1.96 (1.17–3.29)	1.09(0.67–1.78)	0.72	0.83 (0.48–1.44)	0.76 (0.43–1.31)	0.32	0.94 (0.52–1.71)	1.48 (0.99–2.46)	0.13	0.57 (0.14–0.28)	0.84 (0.22–3.30)	0.81	12.82 (5.55–29.62)	6.04 (1.94–18.78)	< 0.001
Risk of wasting (WHZ - ≤ 1 & > −2)	1.63 (1.39–1.91)	1.68 (1.43–1.98)	< 0.001	2.25 (1.44–3.49)	2.54 (1.61–3.99)	< 0.001	1.40 (1.08–1.82)	1.53 (1.18–1.99)	< 0.001	1.68 (1.27–2.21)	1.41 (0.99–2.02)	0.06	0.60 (0.32–1.15)	0.83 (0.44–1.57)	0.57	1.59 (0.64–3.94)	1.65 (0.64–4.26)	0.30
Moderate wasting (WHZ ≤ -2 and > −3)	2.90 (2.41–3.48)	2.78 (2.31–3.36)	< 0.001	8.27 (5.44–12.59)	8.13 (5.27–12.54)	< 0.001	1.71 (1.19–2.44	1.79 (1.24–2.57)	< 0.001	2.04 (1.40–2.97)	2.20 (1.41–3.44)	< 0.001	0.59 (0.17–2.10)	0.76 (0.22–2.69)	0.67	2.35 (0.75–7.34)	1.88 (0.58–6.05)	0.29
Severe wasting (WHZ - ≤ 1 & > −2)	9.09 (7.49–11.02)	7.82 (6.40–9.55)	< 0.001	27.57 (17.71–42.92)	22.81 (14.53–35.80)	< 0.001	3.71 (2.38–5.78)	3.37 (2.14–5.32)	< 0.001	4.34 (2.65–7.10)	5.37 (3.04–9.50)	< 0.001	1.68 (0.47–6.00)	1.78 (0.51–6.30)	0.37	16.30 (6.36–41.80)	9.33 (3.39–25.70)	< 0.001

All *P*-values are for adjusted risk ratios. Adjusted model is corrected for all other variables in the table, plus access to transport and mother’s education level. ^a^ Continuous variable. ^b^ Including children with weight-for-height data in the 101 days preceding death (N = 30)

## Discussion

This is the first study to analyse data collected routinely by an electronic medical records system in West Africa, and is one of only a small number of studies to use primary health care data to assess disease patterns and nutritional status in a sub-Saharan African context [7, 8].

The main strengths of our study lie in the magnitude of our data set, with over 21,000 acute clinic visits over a 5-year period, and the robust electronic capture methodology. For a rural primary care setting, our data are highly complete, with clinic outcome data for 99% of all visits. Diagnoses in our study were solely made by paediatricians or general physicians as opposed to nurse-led clinics which are more common in such settings [16] and although misclassifications may be present [17], the use of ICD-10 coding makes our results easily comparable to other studies.

Infectious diseases formed the majority of the morbidity burden in our study regardless of clinical severity reaffirming the need for focused efforts aimed at both preventing and treating these illnesses [18]. Both severe wasting and severe stunting were associated with an increased risk of death, similar to previous findings [19–21]. Wasting was a strong risk factor for severe illness, overall and for the most common infectious diseases in a dose dependent manner. In the adjusted model, stunting–even when severe– was not associated with an increased risk of severe illness. When estimating the fraction of severe illness attributable to WHZ the inclusion of those at risk of wasting increases this from around one-fifth to almost one-third. Our findings that children at risk of wasting (WHZ ≤ -1 to > − 2) have an almost 70% increased risk of severe illness compared to children who are not classified as at risk (WHZ > -1), indicates that children who would not traditionally be identified as being acutely malnourished are still more vulnerable to severe illness than their better nourished counterparts. This reemphasises the need for a range of actions that promote optimal nutrition in young children, while ensuring that therapeutic measures are in place for those in need.

Community management with ready to use therapeutic food has proven highly effective in treating children with severe wasting [22, 23]. Coverage of therapeutic programmes can however be low, due to programmatic constraints, or a lack of awareness of malnutrition or the therapeutic services in communities [24]. Recent advances in the formulation of therapeutic foods could have important implications for the cost and logistics currently associated with these programme, [25] which may lead to increased provision of these services. The effectiveness of community treatment for moderate wasting is unclear from both programmatic and policy perspectives [26–28]. Currently the routine provision of supplementary foods to children with moderate wasting is not recommended [29].

Often the dietary guidance provided for carers of children with moderate wasting is no different to that of well-nourished children, while they need guidance that is locally adapted and promotes nutritionally adequate diet for their physiological state [30].

A range of efficacious nutrition interventions are available, that when delivered at high coverage could significantly reduce the burden of wasting and stunting [31]. Nutrition educational strategies and mass media interventions can be effective at improving infant and child feeding practices, yet we need to know more about the specific design components of these interventions that deliver the greatest impact [32]. There is also evidence multi-faceted nutrition specific interventions deliver greater benefit over singular focused programmes, [33]. Additionally, to address both the proximal and distal determinants of malnutrition and ensure sustained impact, nutrition interventions need to be implemented in conjunction with other nutrition sensitive public health measures, including social safety nets [34].

Despite a long history of nutritional intervention studies in Kiang West [35] the level of malnutrition in our study population equates to a serious public health problem [36], although this is not dissimilar to the situation in many low-income countries [37, 38]. Our results highlight the importance of early identification of poor nutritional status in children. The collection of multiple anthropometric measurements enabling the assessment of growth velocity rather than one off measurements could be a more effective strategy [39], however in many contexts this approach may prove impractical [40]. This challenge could partly be overcome by the use of simple measurements that caregivers can conduct at home regularly [41]. There is growing evidence for the effect of mHealth interventions [42], and we believe technology innovations, such as an SMS based platform like Rapid Pro [43], could be used for self-reporting and to facilitate focused guidance for caregivers and to enable remote follow up.

As well as identifying specific interventions, striking a balance between the different delivery platforms for nutrition services – health facility, community and out-reaches [44] – with respect to effectiveness for nutrition outcomes and cost remains challenging. However, as a contact point where help is sought for common issues, such as cough and fever, rural clinics present an ideal place where targeted assessment, care and referrals can be provided. This is further supported by the observations that the outcomes of community-wide preventative nutrition supplementation programmes in children have been conflicting, particularly in terms of morbidity, which may relate to a lack of targeting for those most in need [45, 46]. Equally, nutritional supplementation in those with severe illness can also have variable effects on subsequent malnutrition [47] and morbidity [48] supporting targeted intervention at primary health care level. For such an approach to be successful there is a need to build the capacity and knowledge of front line health workers around nutrition [49], to strengthen community structures and build better linkages between primary care and community services [50].

Effective and accurate management of the primary complaint at presentation is however paramount. This study not only highlights the increased risk of severe illness with undernutrition, leading to our call for targeted preventative measures in primary care, but also highlights again the lack of adherence to clinical guidelines [51]. Over two-thirds of all children received antibiotics, including one-third of children with common cold. Inappropriate prescribing of drugs remains a global issue in the era of antimicrobial resistance [51–53]. Interventions aimed at both supply and demand side of prescription medications are required to redress the overuse of these medications, with the identification of local barriers to change important [54].

We acknowledge that our study has a number of limitations. While Kiang West has many similarities to other rural sub-Saharan African settings, the provision of high-quality free health services is unlikely to be one of them, although health and medication provision is also free in other government run health facilities in The Gambia. Information on a number of key factors related to nutritional status were also not available, including exclusive breastfeeding in infants under 6 months of age. Verbal autopsies were not available for the registered deaths and the majority of children who died did not present to the clinic prior to death, limiting our ability to analyse the majority of child deaths. However, our study highlights the need for approaches to reduce neonatal deaths and further examine the impact of improving access to care, especially in the neonatal period and for those furthest from primary care sites. The PAF is based on an assumption of causality between the exposure and outcome, however we recognise that the interactions between nutritional status and infectious disease are not unidirectional, and that our results do not prove causality. Further investigation is required in order to unravel the sequence of events in complex nutrition-infection interactions. [55]

## Conclusion

The association between nutritional status and illness is complex, yet we believe our findings provide a strong case for further investment in improving child nutrition, especially in those at risk or suffering from wasting, as a key strategy for preventing acute disease and promoting child survival. While there are a number of efficacious nutrition interventions, knowledge gaps remain, including specific guidance on the treatment of moderate wasting. Early identification of poor or worsening nutrition status is an important factor for addressing malnutrition, the role of technology in facilitating this is worthy of further exploration. Operational research is also required to strengthen the evidence of how to take proven interventions to the scale required for maximum impact.

## Additional file

Additional file 1: is attached in conjunction with this manuscript as a word file (.doc). The additional file contains six sections as follows. Section 1: Specific search terms used in the literature review. Section 2: Detailed list of variables extracted from the Kiang West Demographic Surveillance Systsem (DSS) and KEMReS databases Section 3: Explanation of severity criteria and cut offs from Rees et al. (2016). Section 4: Clinic outcomes, presenting complaints, diagnoses and prescriptions during 21,278 acute clinic visits by age groups. Section 5:Demographic characteristics of children with missing anthropometric data compared with children not missing data. Section 6: Anthropometric indicators disaggregated by age (< 1 year vs over 1 year). (DOCX 33 kb)

## Abbreviations

aOR: Adjusted Odds Ratio
DSS: Demographic Surveillance System
HAZ: Height for age z-score
ICD: International Statistical Classification of Diseases and Related Health Problems
IQR: Interquartile range
KEMReS: Keneba Electronic Medical Records System
MRC: Medical Research Council
OR: Odds ratio
PAF: Population attributable fraction
PAWS: Paediatric Advanced Warning Score
WHZ: Weight for height z-score
