# Supplementary material for: Nutritional status and disease severity in children acutely presenting to a primary health clinic in rural Gambia
Source: BMC Public Health. 2019 May 30;19:668. doi: 10.1186/s12889-019-6959-y (PMC6543667; doi:10.1186/s12889-019-6959-y)
Supplement: Supplementary file 1 — is attached in conjunction with this manuscript as a word file (.doc). The additional file contains six sections as follows. Section 1: Specific search terms used in the literature review. Section 2: Detailed list of variables extracted from the Kiang West Demographic Surveillance Systsem (DSS) and KEMReS databases Section 3: Explanation of severity criteria and cut offs from Rees et al. (2016). Section 4: Clinic outcomes, presenting complaints, diagnoses and prescriptions during 21,278 acute clinic visits by age groups. Section 5:Demographic characteristics of children with missing anthropometric data compared with children not missing data. Section 6: Anthropometric indicators disaggregated by age (< 1 year vs over 1 year). (DOCX 33 kb) [file 12889_2019_6959_MOESM1_ESM.docx]

**Diagnostic patterns and disease severity in children presenting to a primary health clinic in rural Gambia and association with nutritional status**

**Additional file 1 section 1:**

**Literature review search terms**

(((((primary healthcare data [Text Word]) OR primary care data [Text Word]) OR Primary Health Care [MeSH Terms]) OR Child Health Services [MeSH Terms]) OR electronic health records [MeSH Terms]) AND ((((morbidity profile* [Text Word]) OR health statistics [Text Word]) OR disease profile* [Text Word]) OR epidemiology [MeSH Terms]) AND ((Child, Preschool [MeSH Terms]) OR Infant [MeSH Terms]) AND ((*Africa* [Text Word]) OR Developing Countries [MeSH Terms])

**Additional file 1 section 2:**

**Data extracted from the Kiang West Demographic Surveillance Systsem (DSS) and KEMReS databases for January 1^st^ 2009 to December 31^st^ 2014**

| ***Required data*** | ***Source*** |
| --- | --- |
| Mid-year population | DSS |
| Mid-year population under 5 years | DSS |
| Population <28 days (male and female) | DSS |
| Population >28d - <12 months (male and female) | DSS |
| Population 12-59 months (male and female) | DSS |
| WKNO | KEMRes |
| dVisitDate | KEMRes |
| DateOfBirth | KEMRes |
| Calculated age in mths | KEMRes |
| Gender | KEMRes |
| Village | KEMRes |
| DistToKeneba | KEMRes |
| BirthOrder | KEMRes |
| SameFather | KEMRes |
| SameMother | KEMRes |
| DeadSibling | KEMRes |
| WifeNo | KEMRes |
| FatherNoOfWives | KEMRes |
| MotherAge | KEMRes |
| MotherEducation | KEMRes |
| FatherAge | KEMRes |
| ParentsStillMarried | KEMRes |
| MotherStatus | KEMRes |
| LivingWithMother | KEMRes |
| PresentComplaint | KEMRes |
| Physical examination | KEMRes |
| AppointmentType | KEMRes |
| cDiagCode | KEMRes |
| cDiagName | KEMRes |
| cDrug | KEMRes |
| cActionText | KEMRes |
| cDiagnosticsText | KEMRes |
| IVAntibioticUse | KEMRes |
| nAHome | KEMRes |
| nAReferral | KEMRes |
| nAWhy | KEMRes |
| nAFollowup | KEMRes |
| nATypeFollowup | KEMRes |
| cPatientID | KEMRes |
| dVisitDate | KEMRes |
| nBPSYS | KEMRes |
| nBPDYS | KEMRes |
| nOxygenSaturation | KEMRes |
| nHeartRate | KEMRes |
| nRespiratoryRate | KEMRes |
| nTemperature | KEMRes |
| nCRT | KEMRes |
| COMAscore | KEMRes |
| nBestEyeResp | KEMRes |
| nBestVarbalResp | KEMRes |
| nBestMotorResp | KEMRes |
| nWeight | KEMRes |
| nHeight | KEMRes |
| nHC | KEMRes |
| nMUAC | KEMRes |
| nSFT | KEMRes |
| nEmrgSignNone | KEMRes |
| nObstructedBreath | KEMRes |
| nSevereRespDistress | KEMRes |
| nCentralCyanosis | KEMRes |
| nCirSignShock | KEMRes |
| nCmCnCf | KEMRes |
| nSvrDehydration | KEMRes |
| nPrioritySignNone | KEMRes |
| nUnder2Months | KEMRes |
| nTempHigh | KEMRes |
| nRespDistress | KEMRes |
| nTrauma | KEMRes |
| nPallor | KEMRes |
| nPoisoning | KEMRes |
| nPainsevere | KEMRes |
| nReslessCIlethergic | KEMRes |
| nMalnutrition | KEMRes |
| nOedemaBothFeet | KEMRes |
| nMajorBurn | KEMRes |
| nDairrhoea | KEMRes |
| nClinicalStatus | KEMRes |

**Additional file 1 section 3**

**Summary explanation of the severity criteria and cut offs from: Rees CP, Hawkesworth S, Moore SE, Dondeh BL, Unger SA. Factors affecting access to healthcare: an observational study of children under 5 years of age presenting to a rural Gambian primary healthcare centre. PLoS One. 2016;11(6):e0157790. doi:10.1371/journal.pone.0157790**.

Illness severity was calculated using 5 criteria. For each of these criteria that were fulfilled one point was awarded giving an overall severity score. A cut-off score of 2 or above out of 5 was chosen to indicate severe illness to increase the specificity. The criteria included 1) history and examination findings meeting IMCI criteria for severe illness, 2) if the clinician felt they were acutely ill looking, 3) if they required parenteral treatment, 4) if they required observation or referral, and 5) if they had a positive early warning score of 3+ out of 5.

The criteria included an early warning score, as it is known that triage observations offer a valuable insight into the severity of illness. This study used the Paediatric Advanced Warning Score (PAWS), developed in 2008 [1] in conjunction with the Advanced Paediatric Life Support guidelines. This PAWS scoring system has been validated in an accident and emergency setting in the UK, with a sensitivity of 70% and specificity of 90% for children needing intensive care admission [1]. This system was chosen as the observations needed to calculate the score were available from KEMReS or easily modified. Points were given for abnormal observations and a score of 3 or greater indicates severe illness. One point was given for each entity. The WHO definitions of severe malaria, severe pneumonia and bronchiolitis and severe diarrhoeal illness were adapted using equivalent criteria documented in the KEMReS database. Clinicians documented if the child was ‘acutely illlooking’. Although subjective, this provides a useful overview of how the clinician felt the patient’s condition was. The use of parenteral treatment suggests severe disease, as it is the recommended treatment for severe illness. In severe cases a child would be observed in the clinic observation bay or referred to a hospital. The early warning score was based on observations at triage and was adapted to the setting.

**Reference:**

1. Egdell P, Finlay L, Pedley DK. The PAWS score: validation of an early warning scoring system for the initial assessment of children in the emergency department. Emerg Med J. 2008; 25(11):745–9. doi: 10. 1136/emj.2007.054965 PMID: 18955610

**Additional file 1 section 4:**

**Clinic outcomes, presenting complaints, diagnoses and prescriptions during 21,278 acute clinic visits by age groups.**

|  | **Under 1 (n/visits)** | **1-5 (n/visits)** | **0- 5 (n/visits)** |
| --- | --- | --- | --- |
| **Common presenting complaints during acute visits (n=21,278)** | | |  |
| Fever | 4987/6605  (75.5%) | 10,491/14,673 (71.5%) | 15,478/21,278 (72.7%) |
| Cough | 3765/6605  (57.0%) | 7195/14,673  (49.0%) | 10,960/21,278 (51.5%) |
| Diarrhoea | 1651/6605 (25.0%) | 2065/14,673 (14.0%) | 3716/21,278 (17.5%) |
| Vomiting | 1390/6605 (21.0%) | 1944/14,673 (13.3%) | 3344/21,278 (15.7%) |
| Abdominal pain | 194/6605 (2.9%) | 1194/14,673 (8.1%) | 1388/21,278 (6.5%) |
| Loss of appetite | 328/6605 (5.0%) | 888/14,673 (6.1%) | 1216/21,278 (5.7%) |
| Skin problems | 303/6605 (4.6%) | 588/14,673 (4.0%) | 891/21,278 (4.2%) |
| Headache | 33/6605 (0.5%) | 818/14,673 (5.6%) | 851/21,278 (4.0%) |
| Ear problems | 138/6605 (2.1%) | 437/14,673 (3.0%) | 575/21,278 (2.7%) |
| Chest pain | 87/6605 (1.4%) | 317/14,673 (2.2%) | 404/21,278 (1.9%) |
| **Common diagnoses during acute visits (n=21,278)** | | |  |
| Common cold | 2485/6605 (37.6%) | 5148/14,673 (35.1%) | 7633/21,278 (35.9%) |
| Skin infections | 871/6605 (13.2%) | 2623/14,673 (17.9%) | 3494/21,278 (16.4%) |
| Viral intestinal infections | 1319/6605 (20.0%) | 1576/14,673 (10.7%) | 2895/21,278 (13.6%) |
| Pneumonia | 725/6605 (11.0%) | 1214/14,673 (8.3%) | 1939/21,278 (9.1%) |
| Conjunctivitis | 402/6605 (6.1%) | 673/14,673 (4.6%) | 1075/21,278 (5.1%) |
| Intestinal helminthiasis | 60/6605 (0.9%) | 1001/14,673 (6.8%) | 1061/21,278 (5.0%) |
| Sepsis, unspecified | 172/6605 (2.6%) | 334/14,673 (2.3%) | 506/21,278 (2.4%) |
| Viral infection, unspecified | 149/6605 (2.3%) | 316/14,673 (2.2%) | 465/21,278 (2.2%) |
| Bacterial intestinal infection | 126/6605 (1.9%) | 304/14,673 (2.1%) | 430/21,278 (2.0%) |
| **Common diagnoses during severe illness presentations (n=961)** | | |  |
| Pneumonia | 115/362 (31.8%) | 172/599(28.7%) | 287/961 (29.9%) |
| Sepsis | 71/362 (19.6%) | 117/599 (19.5%) | 188/961 (19.6%) |
| Viral intestinal infections | 91/362 (25.1%) | 92/599 (15.4%) | 183/961 (19.0%) |
| Nutritional marasmus | 38/362 (10.5%) | 43/599 (7.2%) | 81/961 (8.4%) |
| Acute bronchitis | 43/362 (11.9%) | 23/599 (3.8%) | 66/961 (6.9%) |
| **Most common drugs prescribed during acute visits (n=21,278)** | | | |
| Paracetamol | 5622/6605  (85.1%) | 12,425/14,673 (84.7%) | 18,047/21,278 (84.2%) |
| Antibiotics (all) | 4178/6605  (63.3%) | 10,149/14,673 (69.2%) | 14,327/21,278 (67.3%) |
| Amoxicillin | 1651/6605 (25.0%) | 3637/14,673 (24.8%) | 5288/21,278 (24.9%) |
| Oral rehydration solution | 2248/6605 (34.0%) | 2922/14,673 (19.9%) | 5170/21,278 (24.3%) |
| Co-trimoxazole | 1004/6605 (15.4%) | 3246/14,673 (22.1%) | 4250/21,278 (20.0%) |
| Chlorhexidine | 590/6605 (8.9%) | 2151/14,673 (14.7%) | 2741/21,278 (12.9%) |
| **Clinic outcomes during acute visits (n=21,278)** | | | |
| Sent home | 6003/6605  (90.9%) | 13,627/14,673 (92.9%) | 19,630/21,278 (92.3%) |
| Admitted for observation | 387/6605 (5.8%) | 625/14,673 (4.2%) | 1012/21,278 (4.7%) |
| Referred for further investigation or care | 63/6605 (1.0%) | 103/14,673 (0.7%) | 166/21,278 (0.8%) |
| Admitted to nutrition rehabilitation unit | 93/6605 (1.4%) | 171/14,673 (1.2%) | 264/21,278 (1.2%) |
| Unknown | 59/6605 (0.9%) | 147/14,673 (1.0%) | 206/21,278 (1.0%) |

**Additional file 1 section 5:**

**Demographics of children with missing anthropometric data compared with children not missing data**

|  | **Weight-for-Height data** | | **Height-for-age data** | |
| --- | --- | --- | --- | --- |
|  | **Missing data**  **(n=800)** | **Not missing data**  **(n=20,478)** | **Missing data**  **(n=733)** | **Not missing data**  **(n=20,545)** |
| Median age at visit (IQR) | 1.74 (2.96) | 1.68 (2.14) | 1.39 (2.14) | 1.70 (2.16) |
| Percentage of all presentations by girls | 49.1 | 47.4 | 49.0 | 47.4 |
| Median mothers age at visit (IQR) | 27.0 (11.1) | 31.0 (10.5) | 26.6 (8.7) | 31.0 (11.1) |
| Median birth order at visit (IQR) | 2 (3) | 4(4) | 2 (3) | 4 (4) |
| Median distance (kilometre) to clinic (IQR) | 4.6 (7.5) | 5.3 (11.6) | 4.6 (7.5) | 5.3 (11.6) |
| Access to transport to clinic (%) | 74.9 | 54.2 | 72.3 | 54.3 |
| Children with severe illness (%) | 4.62 | 4.51 | 4.09 | 4.5 |

**Additional file 1 section 6:**

**Nutritional status of children during acute clinic visits according to age group.**

|  | ***All acute visits***  ***N=21,278*** | ***Acute visits under 1 year***  ***N=6,605*** | ***Acute visits 1- 5 years***  ***N=14,673*** |
| --- | --- | --- | --- |
| Mean WHZ (SD) | -0.70 (1.20) | -0.47 (1.35) | -0.80 (1.10) |
| All wasting | 2405/20,478 (11.7%) | 722/6340 (11.4%) | 1683/14,138 (11.9%) |
| Moderate wasting | 1923/20,478 (9.4%) | 547/6340 (8.6%) | 1376/14,138 (9.7%) |
| Severe wasting | 482/20,478 (2.4%) | 175/6340 (2.8%) | 307/14,138 (2.2%) |
| Mean HAZ (SD) | -1.21 (1.30) | -0.88 (1.38) | -1.36 (1.30) |
| All stunting | 4389/20,545 (21.4%) | 887/6317 (14.0) | 3502/14,228 (24.6%) |
| Moderate stunting | 3346/20,545 (16.3%) | 648/6317 (10.3%) | 2698/14,228 (19.0%) |
| Severe stunting | 1043/20,545 (5.1%) | 239/6317 (3.8%) | 804/14,228 (5.7%) |
| Mean WAZ (SD) | -1.22 (1.15) | -0.92 (1.28) | -1.35 (1.06) |
| All underweight | 4519/20605 (21.9%) | 1054/6337 (16.6%) | 3465/14,268 (24.3%) |
| Moderate underweight | 3529/20605 (17.1%) | 779/6337 (12.29) | 2750/14,268 (19.3%) |
| Severe underweight | 990/20605 (4.8%) | 275/6337 (4.3%) | 715/14,268 (5.0%) |
